# Performance Analysis in Rugby Union: a Critical Systematic Review

**DOI:** 10.1186/s40798-019-0232-x

**Published:** 2020-01-15

**Authors:** Carmen M. E. Colomer, David B. Pyne, Mitch Mooney, Andrew McKune, Benjamin G. Serpell

**Affiliations:** 1 Research Institute for Sport and Exercise, University of Canberra, Canberra, Australia; 2 Brumbies Rugby, University of Canberra, Building 29, University Drive, Bruce, Canberra, ACT 2617 Australia; 3 Netball Australia, Melbourne, Australia; 4 School of Exercise Science, Australian Catholic University, Melbourne, Australia

**Keywords:** Performance indicators, Tactical analysis, Game analysis

## Abstract

**Background:**

Performance analysis in rugby union has become an integral part of the coaching process. Although performance analysis research in rugby and data collection has progressed, the utility of the insights is not well understood. The primary objective of this review is to consider the current state of performance analysis research in professional rugby union and consider the utility of common methods of analysing performance and the applicability of these methods within professional coaching practice.

**Methods:**

SPORTDiscus electronic database was searched for relevant articles published between 1 January 1997 and 7 March 2019. Professional, male 15-a-side rugby union studies that included relevant data on tactical and performance evaluation, and statistical compilation of time-motion analysis were included. Studies were categorised based on the main focus and each study was reviewed by assessing a number of factors such as context, opposition analysis, competition and sample size.

**Results:**

Forty-one studies met the inclusion criteria. The majority of these studies measured performance through the collection and analysis of performance indicators. The majority did not provide context relating to multiple confounding factors such as field location, match location and opposition information. Twenty-nine performance indicators differentiated between successful match outcomes; however, only eight were commonly shared across some studies. Five studies considered rugby union as a dynamical system; however, these studies were limited in analysing lower or national-level competitions.

**Conclusions:**

The review highlighted the issues associated with assessing isolated measures of performance, lacking contextual information such as the opposition, match location, period within match and field location. A small number of studies have assessed rugby union performance through a dynamical systems lens, identifying successful characteristics in collective behaviour patterns in attacking phases. Performance analysis in international rugby union can be advanced by adopting these approaches in addition to methods currently adopted in other team sports.

## Key Points

- Rugby performance analysis continues to rely heavily on isolated measures of performance, such as performance indicators, without providing context to confounding factors such as opposition behaviour, pitch location, period within match and venue location.
- Some studies have investigated team behaviour in rugby union; however, to facilitate a better understanding of group behaviour in international rugby, a dynamical systems analysis approach at an elite level is recommended.
- Within and between team interactions have been measured in other sports including football and basketball. Rugby performance analysis may benefit from adopting strategies employed by these sports in order to gain a better understanding of team properties and the patterns that characterise their coordination.

## Background

Performance analysis in team sports allows coaches to objectively assess the performance of the team while identifying their oppositions’ strengths and weaknesses, and opportunities to exploit these in competition. To do this effectively requires a comprehensive analysis of individual and collective actions, to provide objective summaries of game activities during competition [1]. There has been an exponential growth in performance analysis research over the last two decades, largely a consequence of the advancement and availability of computer and video technology. Broadly, performance analysis involves an objective assessment of documented behaviours recorded in a discrete sequential manner containing information on ‘what’, ‘who’, ‘when’ and ‘where’ the behaviours occurred. Behaviours are typically recorded through annotation software; however, advancements in video capture technologies are allowing player position information to be analysed with associated behaviours to provide a more meaningful understanding of game behaviours. This development has contributed considerably to our understanding the performance requirements in elite-level competition. However, fundamental issues remain in the questions underpinning the research in the field; the cause-and-effect-based observations inherently assume linear relationships to predict and control match outcome. For example, the direction and scope of the research in rugby union has primarily explored a single or a combination of action variables (performance indicators) deemed relevant to successful outcomes such as possession and tackle success [2]. Furthermore, the analysis of these performance indicators has primarily only focused on discrete, descriptive and comparative statistics. Other common research topics have simply studied technical and physical requirements during specific periods or game events, such as peak running intensities [1, 3, 4]. Thus, this type of research assumes human behaviour is causal, measurable and thus predictable.

A further limitation to much of the research on performance analysis in rugby is that there is a lack of evidence surrounding the implementation of this work into everyday practice by coaches and practitioners. The apparent limited influence is potentially due to an absence of consensus between practitioners and scientists, and the information that drives actions and implementation. Performance analysis research is commonly composed by researchers, directing methods and structuring studies, potentially neglecting the applicability and utility of the research findings. Developing the field of performance analysis in rugby needs collaboration between scientists and practitioners to improve the ability of science to influence practice. Bridging the theory-to-practice gap may require developing an applied research model that describes rugby performance in an integrated manner.

To overcome the current methods beset by various issues, it seems pertinent to understand rugby performance as a complex dynamical system. In this sense, the patterns of game behaviour emerge from the self-organising interactions between players operating within task, and environmental and physical constraints [5]. A corollary to this is that rugby performance is highly complex and requires players to perform coordinated tactical behaviours and high-intensity movements with adept technical proficiency, making it difficult to reduce game analysis to isolated measures of performance. Therefore, there is a clear need for performance analysis to reflect and capture this complexity and create a global understanding of performance.

This paper systematically reviews the literature to describe the state of rugby union performance analysis, highlighting the various methods of analysis and exploring variables used to assess performance. We then conclude with some recommendations for future research drawing upon research from Association Football (football [soccer]) as a means of envisaging where the field of rugby could evolve to in the future.

## Methods

A systematic review of the relevant literature was conducted according to the Preferred Reporting Items for Systematic Reviews and Meta-analyses (PRISMA) guidelines. The SPORTDiscus electronic database was searched on 8 March 2019 for relevant articles published between 1 January 1997 and 7 March 2019 using the following search terms:

Rugby AND “collective behav*” OR “tactic* analysis” OR “tactic* performance” OR “tactical indicator*” OR “performance indicator*” OR “performance analysis” OR “notational analysis” OR “game analysis” OR “observational analysis” OR “Pattern* of play” OR “dynamic* system” OR “tactic* behave*” OR “neural network” OR “system* think*” OR “performance model*” OR “player selection” OR “player evaluation” OR “game statistics”.

The inclusion criteria were as follows: included relevant data on tactical performance, time-motion analysis, such as assessments of team movement patterns in relation to time; participants included professional adult male rugby players; the sport analysed was 15-a-side rugby union; and articles were published in English. Articles were limited to journal articles where the full text was available. Studies were excluded if they included females; involved males under the age of 18; analysed rugby league or 7-a-side rugby union; were a conference abstract or doctoral thesis; and did not include relevant data for the study. Major research topics of game analysis that emerged from the detailed analysis were identified and the studies grouped accordingly: performance indicators, attack and defence. Research topics were decided upon by authors deeming the majority of the observations included (a) variables relating to the attacking team; (b) variables relating to the defensive team; or (c) predominantly involved the assessment of performance indicators. Successful and unsuccessful match outcomes were defined as match won and lost, respectively.

### Quality of Studies

Quality of studies was not assessed based on a recognised classification method as the nature of the research valued observational, tactical studies. Therefore, as no experimental studies were included, Delphi, PEDro or Cochrane was not utilised as scales of evaluation. All 41 articles outlined in Table 1 were assessed for suitability and evaluated by the panel of authors prior to inclusion. All studies had to meet every item on the criteria list to be included in the analysis.

**Table 1:** A description of the reviewed studies

Reference	Competition	Focus	Number of events analysed	Opposition analysis
Boddington and Lambert [1]	2003 Rugby World Cup	Attack	35 try scoring observations from 1 team	No
Laird and Lorimer [6]	2003 Six Nations, Tri Nations and Argentina	Attack	152 tries from 32 matches	No
Sayers and Washington-King [7]	2003 Super 12 Rugby Competition	Attack	48 matches from 6 teams	No
van Rooyen and Noakes [8]	2003 Rugby World Cup	Attack	25 matches from 4 teams	No
Sasaki et al. [9]	2003-2005 Japanese Top League	Attack	198 matches	No
Wheeler and Sayers [10]	2006 Super 14 Rugby Competition	Attack	1372 ball carries from 7 matches	Yes
Wheeler et al. [11]	2006 Super 14 Rugby Competition	Attack	1372 ball carries from 7 matches	Yes
Diedrick and van Rooyen [12]	2007 Rugby World Cup	Attack	47 line breaks from 11 matches	No
Lim et al. [13]	2006, 2007 and 2008 Super 14 Rugby Competition	Attack	117 observations from 3 teams	No
van Rooyen [4]	2011 Six Nations, Tri Nations and Rugby World Cup	Defence	48 matches	No
Hendricks et al. [14]	2010 Super 14 Rugby Competition	Defence	2394 tackle events from 21 matches	Yes
Wheeler et al. [15]	2011 Super Rugby Competition	Defence	8563 ruck contests from 60 matches	Yes
Bracewell [16]	2000 Super 12 Rugby Competition	Attack and defence	13 matches	No
Jones et al. [17]	2002–2003 season of a Northern Hemisphere professional rugby competition	Attack and defence	20 matches	No
James et al. [18]	2001–2002 season of a Northern Hemisphere professional rugby competition	Attack and defence	21 matches from 1 team	No
Prim et al. [19]	2005 Super 12 Rugby Competition	Attack and defence	9 matches from 5 teams	No
Rooyen et al. [20]	2003 Rugby World Cup	Attack and defence	26 matches from 4 teams	No
Jones et al. [1]	2003–2004 season of a Northern Hemisphere professional rugby competition	Attack and defence	10 matches from 2 teams	No
Lim et al. [21]	2006, 2007 and 2008 Super 14 Rugby competition	Attack and defence	117 observations from 3 teams	No
Ortega et al. [22]	2003-2006 Six Nations Tournament	Attack an defence	58 matches	No
Van den Berg and Malan [23]	2006 Super 14 Rugby Competition	Attack and defence	185 matches	No
van Rooyen et al. [24]	2007 Rugby World Cup	Attack and defence	5635 rucks from 48 matches	No
Vaz et al. [25]	2003–2006 World Cup, Six Nations, Tri Nations and Super Rugby competitions	Attack and defence	224 matches	No
Correia et al. [26]	2007/2008 season of a Northern Hemisphere professional rugby competition	Attack	22 observations from 5 matches	No
Correia et al. [27]	2007/2008 season of a Northern Hemisphere professional rugby competition	Attack	13 observations	Yes
Hughes et al. [28]	2011 Rugby World Cup	Attack and defence	26 matches	Yes
Bishop and Barnes [29]	2011 knockout stages of the Rugby World Cup	Attack and defence	8 teams	No
Bremner et al. [30]	Two seasons of a professional Australian Rugby Union team	Attack and defence	65 matches	No
Gaviglio et al. [31]	One season of a Northern Hemisphere professional rugby team	Attack and defence	31 matches	No
Rodrigues and Passos [32]	2010/2011 season of a Northern Hemisphere professional rugby competition	Attack and defence	15 observations from 3 matches	Yes
Kraak and Welman [33]	2010 Six Nations Championship	Attack and defence	1479 rucks from 15 matches	Yes
Schoeman and Coetzee [34]	2005–2007 Super 14 competitions, Tri-nations and International test matches	Attack and defence	18 matches	No
Smart et al. [3]	2007–2008 New Zealand national provincial, professional Super 14 and international level competitions	Attack and defence	510 players from 296 matches	No
Croft et al. [35]	2013 New Zealand national provincial competition	Attack and defence	76 matches	
Vahed et al [36]	2007 and 2013 South African Currie Cup tournament	Attack and defence	70 matches	No
Hughes et al. [37]	Knockout stages of the 2015 Rugby World Cup	Attack and defence	8 matches	No
Schoeman et al. [38]	2014 Super Rugby competition and 2014 South African Currie Cup tournament	Attack and defence	60 matches	No
Watson et al. [39]	2014 Super Rugby competition and 2014 South African Currie Cup tournament	Attack and defence	313 matches	No
Sherwood et al. [40]	2015 Super Rugby Season	Attack and defence	260 scrums	Yes
Bennett et al. [41]	2016–2017 English Premiership Rugby Union season	Attack and defence	132 matches from 12 teams	Yes
Coughlan et al. [42]	2017 Super Rugby Competition	Attack	943 tries from 135 games consisting of 18 teams	No

## Results

**Fig. 1:**
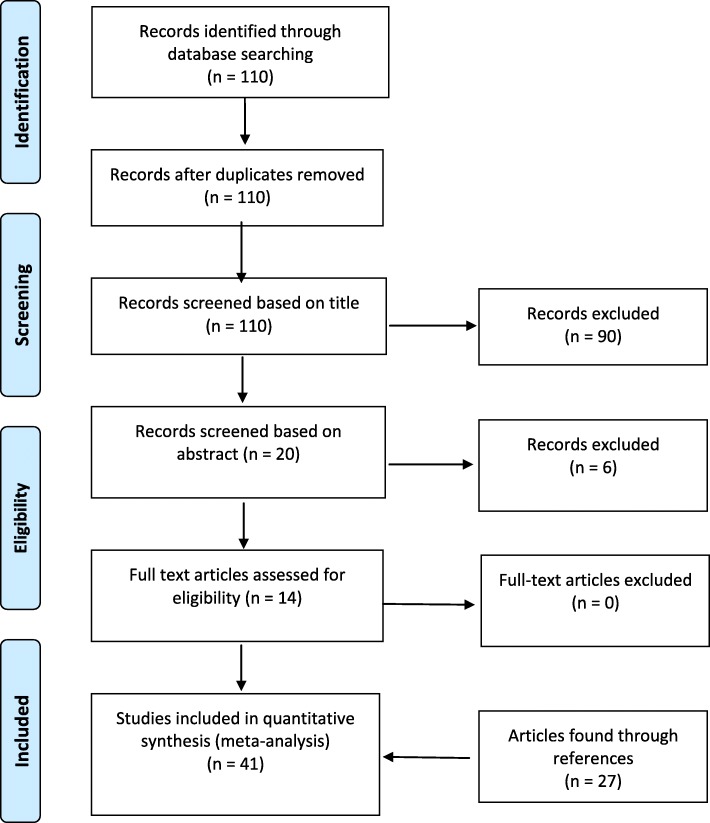
PRISMA (Preferred Reporting Items for Systematic Reviews and Meta-Analyses) flow diagram summarising the search results

The initial search revealed 110 papers. Titles were screened by two members of the research team for inclusion/exclusion criteria. Ninety articles were then removed. The abstracts of the 20 remaining articles were then read by the same two members of the research team where a further six articles were removed, resulting in 14 articles remaining for review. After reading the full texts, all papers were deemed suitable for review. An iterative reference check was then performed of all eligible papers and any commonly cited papers were also included and a further 27 papers were identified. In total, 41 papers were included for discussion (Fig. 1).

### Data Organisation

The following variables were analysed in each study: (1) competition level (including geographic location); (2) main focus; (3) key performance indicators (including selection process, successful indicators and operational definitions); (4) contextualised variables; (5) opposition analysis; and (6) studies that used a dynamical systems approach (Tables 1, 2, and 3).

**Table 2:** A summary of performance indicators related to success

Successful performance indicators	Level	Study
Lineout success on opposition ball	Super Rugby, international	Hughes et al. [37]; Jones et al. [17]
Tries scored	Super Rugby, international, professional domestic	Jones et al. [17]; Watson et al. [39]
Points scored	International, domestic professional, Super Rugby	Watson et al. [39]; Ortega et al. [22]
Points scored (when possession starts in the opposition 22-m area)	International, Super Rugby, professional domestic	Watson et al. [39]; van Rooyen [4]; Laird and Lorimer [43]
Points scored (when possession starts outside the opposition 22-m area)	International, Super Rugby, professional domestic	Watson et al. [39]; van Rooyen [4]
Conversions	International, Super Rugby, professional domestic	Watson et al. [39]; Ortega et al. [22]
Successful drop (goal)	International	Ortega et al. [22]
Successful penalty goals	International, Super Rugby, professional domestic	Watson et al. [39]
Line breaks	International	Ortega et al. [22]
Possession kicked	International, Super Rugby	Hughes et al. [37]; Ortega et al. [22]; Vaz et al. [25]
Tackles completed	International, Super Rugby	Ortega et al. [22]; Vaz et al. [25]
Turnovers won	International, Super Rugby	Ortega et al. [22]; Vaz et al. [25]
Rucks (-)	Super Rugby	Vaz et al. [25]
Passes (-)	Super Rugby	Vaz et al. [25]
Mauls won	Super Rugby	Vaz et al. [25]
Errors (-)	International, Super Rugby, professional domestic	Watson et al. [39]; Vaz et al. [25]
Conceded penalties (between 50 m and opposition 22 m)	International	Bishop and Barnes [29]
Kicks out of hand	International, Super Rugby, professional domestic	Watson et al. [39]; Bishop and Barnes [29]
Quick rucks (in the 0–20- and 60–70-mintime interval)	Super Rugby	Bremner et al. [30]
Territory (entries in the opposition 22 m, in the 0–20-min time interval)	Super Rugby	Bremner et al. [30]
Gain line +	Super Rugby	Bremner et al. [30]
Gain line +P	Super Rugby	Bremner et al. [30]
AggPI = (tackle wins + ball carries and dominant + clear-out: effective) + (contacts/2)	Professional domestic	Gaviglio et al. [31]
% total tries	International, Super Rugby, professional domestic	Watson et al. [39]
% possession	International, Super Rugby, professional domestic	Watson et al. [39]
Unopposed runs	International, Super Rugby, professional domestic	Watson et al. [39]
Kicks (relative)	Professional domestic	Bennett et al. [41]
Clean breaks (relative)	Professional domestic	Bennett et al. [41]
Average carry metres (relative)	Professional domestic	Bennett et al. [41]

(-): less than unsuccessful teams; “Gain line +”: crossing the opposition gain line; “Gain line +P”: not defined by the authors; AggPI: aggression performance indicator (tackle wins + ball carries and dominant + clear-out: effective) + (contacts/2)

**Table 3:** A summary of performance indicators

Reference	Number of performance indicators listed under themes	Operational definitions	Context	Performance indicators selection
Bracewell [16]: 2000 Super 12 Rugby Competition	Attack (*n* = 20), defence (*n* = 8), other (*n* = 3)	No	N/A	Undisclosed
Jones et al. [17]: 2002–2003 season of a Northern Hemisphere professional rugby competition	Attack (*n* = 8), defence (*n* = 4), set piece (*n* = 4), other (*n* = 6)	No	Field location, match outcome	Compiled by research team then content validated by professional coaches
Laird and Lorimer [6]: 2003 Six Nations, Tri Nations and Argentina	Attack (*n* = 4)	Full operational definitions provided	Field location, period during match	Selected by research group based on previous research
James et al. [18]: 2001–2002 season of a Northern Hemisphere professional rugby competition	Attack (*n* = 14), defence (*n* = 3), set piece (*n* = 2), other (*n* = 2)	No	No	Identified and evaluated by researchers
Prim et al. [19]: 2005 Super 12 Rugby Competition	Attack (*n* = 4), defence (*n* = 5)	Full operational definitions provided	Number of players, match phase	Obtained through a panel of elite coaches and analysts
Rooyen et al. [20]: 2003 Rugby World Cup	Attack (*n* = 6), defence (*n* = 7)	No	Period during match, field location	Simple match descriptors displayed on the International Rugby Board’s (IRB) official website
Jones et al. [1]: 2003–2004 season of a Northern Hemisphere professional rugby competition	Attack (*n* = 4), defence (*n* = 2), set piece (*n* = 4), other (*n* = 2)	No	No	Developed in collaboration with authors and two elite teams’ performance analysts
Lim et al. [21]: 2006, 2007 and 2008 Super 14 Rugby competition	Attack (*n* = 13), defence (*n* = 6), set piece (*n* = 8), other (*n* = 7)	Full operational definitions provided	No	Developed in conjunction authors and coaching staff from an undisclosed Super Rugby team
Ortega et al. [22]: 2003–2006 Six Nations Tournament	Attack (*n* = 14), defence *n* = (8), set piece (*n* = 4), other (*n* = 1)	No	Match outcome	Standard statistics available through governing body website
Van den Berg and Malan [23]: 2006 Super 14 Rugby Competition	Attack (*n* = 12), defence (*n* = 2), set piece (*n* = 2), other (*n* = 1)	No	Team ranking	Standard statistics available through sport analysis company
Vaz et al. [25]: 2003–2006 World Cup, Six Nations, Tri Nations and Super Rugby competitions	Attack (*n* = 9), defence (*n* = 3), set piece (*n* = 4), other (*n* = 2)	No	Match outcome	‘Specialised data centres’
Lim et al. [13]: 2006, 2007 and 2008 Super 14 Rugby Competition	Attack (*n* = 13), defence (*n* = 6), set piece (*n* = 8), other (*n* = 7)	Full operational definitions provided	No	Developed in conjunction authors and coaching staff from an undisclosed Super Rugby team
Hughes et al. [28]: 2011 Rugby World Cup	Attack (*n* = 10), set piece (*n* = 2), other (*n* = 2)	No	Competition ranking	Standard statistics available through governing body website
Bishop and Barnes [29]: 2011 knockout stages of the Rugby World Cup	Attack (*n* = 5), defence (*n* = 2), set piece (*n* = 1), other (*n* = 2)	No	Field position, match outcome	Developed by researchers after a complete review of the literature
Bremner et al. [30]: 2 seasons of a professional Australian Rugby Union team	Attack (*n* = 10), defence (*n* = 10)	No	Period during match	Developed by researchers after a complete review of the literature, then content validated by coaches and analysts
Gaviglio et al. [31]: 1 season of a Northern Hemisphere professional rugby team	Attack (*n* = 1), defence (*n* = 1)	Full operational definitions provided	Match outcome	Selected in conjunction with the team analyst and coaching staff
Smart et al. [3]: 2007–2008 New Zealand national provincial, professional Super 14 and international-level competitions	Attack (*n* = 10), defence (*n* = 2), other (*n* = 1)	Full operational definitions provided	No	Selected by research group based on previous research
Vahed et al. [36]: 2007 and 2013 South African Currie Cup tournament	Attack (*n* = 11), defence (*n* = 5), set piece (*n* = 2), other (*n* = 4)	Full operational definitions provided	Period during match	Undisclosed
Hughes et al. [37]: knockout stages of the 2015 Rugby World Cup	Attack (*n* = 8), defence (*n* = 1), set piece (*n* = 2), other (*n* = 3)	No	Field location	Selected by research group based on previous research
Schoeman et al. [38]: 2014 Super Rugby competition and 2014 South African Currie Cup tournament	Defence (*n* = 1), set piece (*n* = 4), other (*n* = 3)	No	Level of competition	Third-party company
Watson et al. [39]: 5 domestic and international competitions	Attack (*n* = 22), defence (*n* = 5), set piece (*n* = 4), other (*n* = 3)	No	Level of competition	Selected by research group based on previous research. Only performance indicators found to be statistically significant at the team level were selected
Bennett et al. [41]: 2016–2017 English Premiership Rugby Union season	Attack (*n* = 6), defence (*n* = 4), set piece (*n* = 2)	No	No	Undisclosed

*N/A* not applicable

### Year of Publication and Competition

**Fig. 2:**
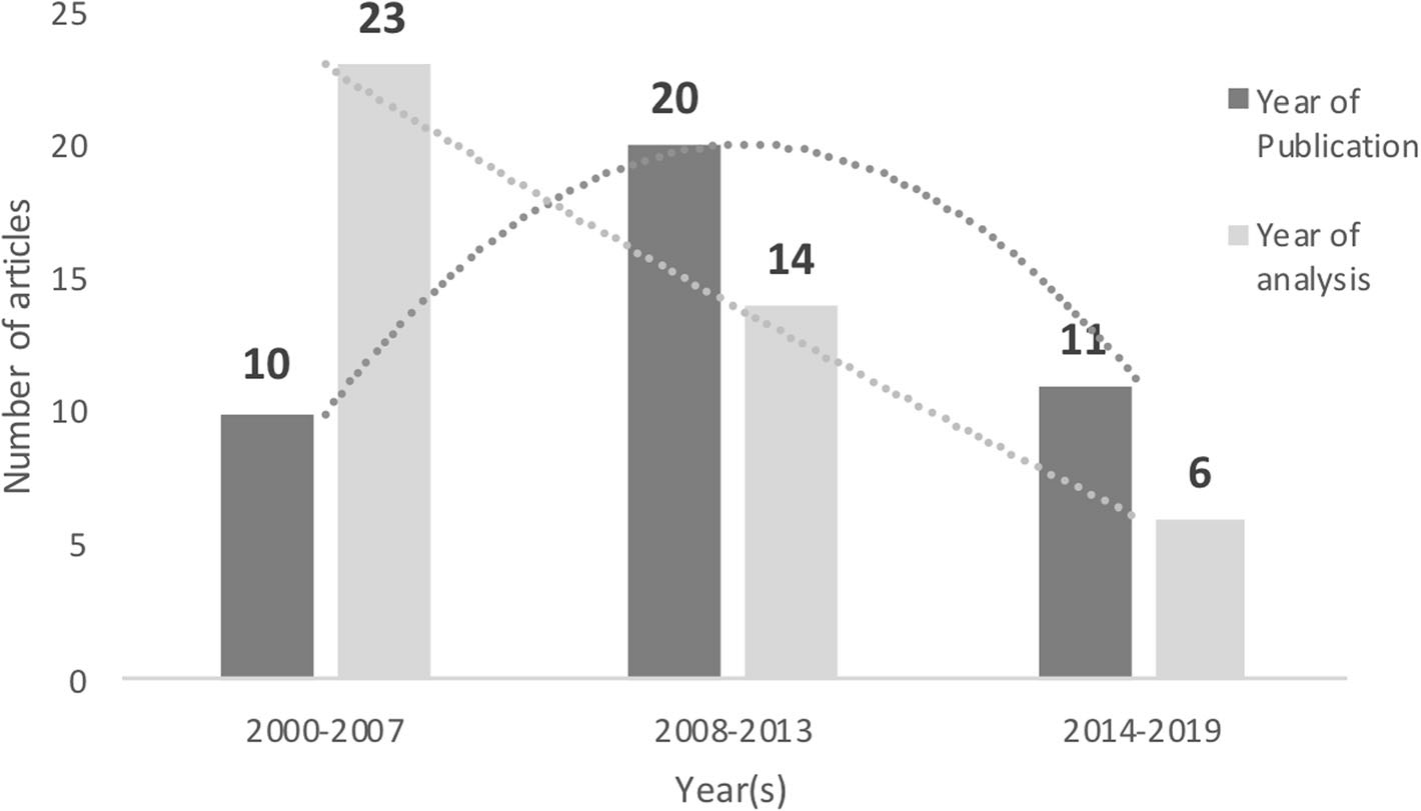
Distribution of articles by years of publication and years of analysis

The 41 articles reviewed are presented in Table 1. In short, the articles were grouped into 5-year intervals by year of publication which resulted in an inverse parabolic curve representation of publication dates where 49% of the articles were published between 2008 and 2013 (Fig. 2). When articles were grouped into year of data collection and analysis, ~ 50% of the articles analysed data from games played between 2000 and 2008 (Fig. 2). Following this period, there has been a linear decrease in the collection of data for publication in rugby union performance analysis research.

The year with the most publications was 2013 (*n* = 5) (Table 1), followed by 2010 (*n* = 4). The year of data collection and analysis was additionally considered important when interpreting results as game styles may have evolved from the time data were collected to the date of publication (Fig. 2). The period from 2003 to 2007 was the most heavily investigated time interval, with 2003 representing the most popular year of analysis (Table 1). Multiple competitions at various levels were investigated in the reviewed studies, ranging from elite domestic leagues to the Rugby World Cup. The most recurrently investigated competition was the Super Rugby Championship with 2006 representing the most frequently investigated season. The 2003 Rugby World Cup was the most investigated World Cup year, followed by 2007 and 2011.

### Analysis of Opposition and Context

The majority of the articles did not include the opposition in their analysis. The ~ 20% that considered the opposition included events such as ball carries (Table 1), tackles, rucks, scrums and performance indicators. Seventy-one percent of the articles that investigated performance indicators contextualised the data (Table 3). Variables were contextualised to field location, match outcome, period during match, numbers of players involved, match phase, team ranking and competition level. Of the 22 articles that contextualised their measures of performance, only five accounted for multiple contextual variables.

### Sample Size and Events

The sample sizes ranged from seven matches to 313 matches, with a mean number of 67 match observations (Table 1). Analysis of individual events ranged from 35, when try scoring incidences were explored, to 8563 ruck contests. The events analysed included ball carries, line breaks, tackles, ruck contests, try scoring observations and scrums. Ruck contests were the most commonly investigated individual events, totalling 15,677 individual events analysed across three studies.

### Performance Indicators

A total of 392 performance indicators were identified across the reviewed articles (Table 3). Performance indicators were classified as either attack (*n* = 204); defence (*n* = 85); set piece (*n* = 53); or other (*n* = 50). Variables related to attack were the most frequently assessed measures of performance, followed by those related to defence.

Understanding the genesis of performance indicators might serve as a starting point for developing valid sets of quantitative tactical indicators. Therefore, the method utilised to select variables related to performance was also considered important. The method of selection utilised by the investigators included the following: a collaboration with investigators and coaches and/or experts; those selected solely by the research group; those sourced from a third-party company; and those where the method of selection was not stated.

Providing a detailed description of each performance indicator is essential to maintain transparency when measuring performance-related variables. These operational definitions allow the shared understanding of the variables used ensuring their meaning is unambiguous and understood [43]. Only seven articles provided full operational definitions, while the remaining 15 provided no definitions for the variables investigated (Table 3). Additionally, the majority of the articles that provided full operational definitions developed these in collaboration with coaches and/or experts.

Indicators linked to successful performance are displayed in Table 2. Across the articles investigating performance indicators, 29 variables differentiated between successful and unsuccessful match outcomes. Possession kicked was positively related to performance in three separate studies [22, 25, 37] at the international and Super Rugby level of competition. The second most frequently observed variables were lineout success on opposition ball; tries scored; points scored (including when possession starts in the opposition 22 m area); conversions; tackles completed; turnovers won; and kicks out of hand (Table 2).

## Discussion

The purpose of this literature review was to describe the state of rugby union performance analysis, highlight the various methods of analysis and explore variables used to assess performance. We have revealed that in the last two decades of rugby research, the approach to describing performance has remained largely unchanged. Investigations into successful performance typically continue to rely on univariate measures of performance, reducing performance to singular values (Table 3). In fact, 22 of the 41 studies retrieved focused on descriptive and comparative statistics and often lacked context. Confounding factors such as match venue, officials, weather and the nature of the opposing team have all been suggested to influence team performance, yet are rarely considered in the majority of the research [17]. This level of information details the origin of the data and arguably allows for more meaningful interpretations. Critical information may, therefore, be lost if performance-related variables are not contextualised and measured while considering these factors [44]. For instance, a major confounding factor is the opposition team yet only eight of the articles retrieved considered the opposing team in the analysis [10, 11, 14, 15, 28, 32, 33, 40]. More than half of the articles investigated successful and unsuccessful measures of performance by quantifying performance indicators over entire competitions. Although this approach is useful as a means to increase the number of data, this level of analysis ignores the variation in playing style over each match and typically lacks consideration of the influence of opposition. Ignoring data from the opposition will likely distort any relationships present [41], particularly when one considers that various studies included data over multiple competitions [3, 4, 6, 25, 38] as well as over several seasons [9, 21, 22, 25, 30, 34] potentially misrepresenting performance outcomes. One paper examined the efficacy of two methods of data analysis to predict match outcomes [41]; isolated performance indicators, considering only the isolated data from a single team, were compared to a descriptive conversion method by calculating the differences between each team’s data for each individual match. That study showed match outcomes were better predicted by relative data sets. Relative predictors of success included an effective kicking game, ball carrying abilities and not conceding penalties when the opposition are in possession.

Although the majority of the studies included contextualised results, it should be noted that some research included contextual information from multiple confounding factors such as pitch location, match period and team ranking. For example, a study of effective strategies at the ruck in the 2010 Six Nations Championship accounted for team ranking, pitch location and number of players involved [33]. The results indicated greater success in regaining possession with a higher ratio of defenders to attackers in ruck situations. Similarly, pitch location and the timing of ruck strategies influenced the outcome of ball possession in the 2011 Super Rugby competition [15]. Defending teams were more likely to turnover possession using an early counter ruck strategy in the wide attacking channels. Conversely, a jackal (a player on the defending team competing for the ball using his hands after a tackle was made but prior to the formation of a ruck) was the most effective strategy in the central field areas. Another study identified quick rucks within the first 20 min and within the 60–70 min time interval had the largest positive effect on match outcome [30], whereas slow rucks had the largest negative effect on winning a match, regardless of the time interval. These results highlight the importance of contextualising performance indicators, as game tactics may need to be adapted depending on the field location, time interval and ruck strategy employed.

Applying the outcome from research using simple, descriptive and isolated variables without consideration of confounding variables is problematic in tactical preparation. For example, set piece tries discriminated between successful and unsuccessful teams [28]; however, without contextual information such as score differential, weather conditions, pitch location or team ranking, little inference can be made regarding how or why behaviours occurred. One study [14] investigating defending strategies in tackle contact events which considered the playing situation, defensive characteristics and phase outcomes bore some insights into effective defensive processes such as defensive speed, field location and period within a match. This study demonstrated that the period of the match and the distance of the contact event in relation to the previous phase are key variables that predict the likelihood of a successful phase outcome. In a practical sense, teams execute different lineout plays depending on the field location (i.e. 5, 6, 7 man; they may play off the top or maul). They may also be more reluctant to throw the ball to the back of the lineout in poor weather conditions. On this basis, set piece selection is commonly dependent on context and, therefore, it is important to consider these factors when assessing performance indicators. Furthermore, analysing the performance of a team assumes that the behaviours in one game will provide insights into future performance in subsequent matches. The fundamental issue is that game behaviours may only specifically represent the performance of a team at the time the data were captured [45].

### Performance Definitions and Indicators

Over 300 performance indicators were identified across 22 studies (Table 3). Interestingly, only 29 were identified as related to successful performance. International tests demonstrated 14 variables (Table 2) discriminating winning and losing teams including higher points scored, kicks, turnovers and penalties conceded between the opposition's 50- and 22-m line. In regional-level competitions, such as Super Rugby in the Southern Hemisphere, 25 variables were identified as successful indicators of performance including a greater number of metres gained, kicks out of hand, line breaks and percentage tackles made compared to losing teams. To illustrate differences in styles of play at different levels of competition, performance indicators that discriminated between winning and losing teams in international test matches and Super Rugby games were investigated [25]. Winners of Super Rugby games kicked more possessions, made more tackles, completed more passes and made less errors. No performance indicators were able to discriminate between winners and losers in international test matches played during 2003 and 2006 when only close matches were investigated (< 15 points difference) [22]. In contrast, another investigation of international games in the same time period showed that winning teams had higher points scoring-related statistics, turn overs and kicks and were more successful at set piece [22]. This discrepancy in outcomes may be a function of close games potentially being played by two opposing high-quality teams, demonstrating similar levels of performance behaviours. This continues to highlight the importance of contextualising performance indicators as vital information is likely to be lost when confounding factors are not considered.

There is typically a lack of transparency in the operational definitions used to describe and analyse rugby performance. Twenty-two retrieved articles quantified performance using performance indicators; however, only 7 actually defined the variables analysed. Furthermore, of the 22 articles, only 16 were explicit about the process of selecting the indicators used. The selection process included expert opinion and research group [1, 17, 21], commonly available statistics by a third-party company [22, 23, 25, 28, 38] and those selected solely by the research group [3, 18, 29, 39] (Table 3). The method used when selecting performance indicators in the remaining articles was undisclosed. Challenges may arise given a lack of clarity (i.e. lack of definitions or objectivity when selecting performance indicators) when comparing or replicating investigations, making it difficult to advance the body of research and for coaching staff to implement the suggested practices. However, a summary of the research and performance indicators relevant to successful performance can provide useful insights.

As mentioned earlier, performance indicators provide an overview of certain events that may contribute to and predict successful performance. However, isolated performance indicators do not consider the opposition, nor do they account for unpredictability and inherent match specificity. For example, game behaviours tend to be inconsistent and performance indicators will most likely be influenced by player-opponent interactions. It is therefore unlikely that a complex, dynamic game such as rugby can be represented by isolated measures of frequency data.

### Evolution of Performance Assessment

Studies relating to attack are more common than investigations into defence (Table 1). Topics such as try scoring, possession duration and ball carries were investigated in relation to the attacking team, whereas tackle contest events and rucks were detailed as measures of defence. Most studies analysing performance indicators investigated both attack and defence situations. Specific investigations into defensive strategies only appeared from 2013 most likely related to rule changes [36] favouring the defensive team during breakdown situations.

To accommodate changing game styles, rule changes were introduced in rugby during 2007 and 2013 expediting the speed of play to increase appeal and competitiveness [36, 46]. The period prior to, during and thereafter should be considered and compared, understanding that successful performance indicators prior to 2007 may not be relevant thereafter. For example, amendments to laws surrounding the ruck led to a decrease in players involved in ruck situations [19]. Teams are instead favouring committing more players to the defensive line in preparation for subsequent phases. As a result, game actions have increased due to the added pressure on attacking teams to expedite the speed of play [36].

Between 2004 and 2007, winning teams won more lineouts on the opposition's throw, scored more tries, had greater metres gained, kicks out of hand, line breaks and percentage tackles made in international, Super Rugby and professional domestic competitions [17, 22, 23]. Successful teams also had higher points scored, conversions, successful drop goals, mauls won, line breaks, possession kicked, tackles completed and turnovers won. In contrast, losing teams lost more scrums and lineouts. Following this epoch, between 2007 and 2013, winning teams conceded more penalties between 50 m and opposition 22 m, and had more total kicks, including kicks out of hand, than losing teams. After 2013, variables likely to result in winning included higher average carry metres, clean breaks made and kicks made relative to the opposition in a professional domestic league. Negative outcomes were more likely when teams conceded penalties while the opposition was in possession. Data were considered in relation to the opposition rather than isolated data of each team considered discretely [41]. Isolated methods of analysis indicated winning teams missed less tackles in the Super Rugby competition [38]. Analysis of knockout stages of the Rugby World Cup, however, indicated that winning teams kicked a greater percentage of possession in the opposition 22–50 m and won more lineouts on the opposition ball [37], suggesting that successful test rugby may require a territory style of play. Performance indicators investigated were inconsistent across the studies, making it difficult to compare and assess the relevance and impact of key attacking and defensive variables. As such, although points scored were unrelated to match outcome post 2013 [41], it is problematic to suggest that point scoring is not important in rugby performance.

Factors such as competition location may rationalise the differing game styles observed. Approximately 20% of studies reported on Northern Hemisphere teams known to have a different style of play to [47] to Southern Hemisphere competitions. Southern Hemisphere teams tend to exhibit higher overall ball-in-play periods resulting in more game actions and injuries due to greater game continuity [47]. Additionally, ~ 40% of articles investigated teams competing in international competitions (Table 1) and 13% included data sets from multiple competitions, possibly decreasing their relevance as some information may be missed given the loss of contextual information [48]. Maintaining the integrity of each individual match when using the established descriptive conversion method of analysis, which considers all performance indicators in relation to the opposition, is preferred [41].

In summary, studies of performance analysis in rugby often show methodological shortcomings regarding the genesis of performance indicators and selection process, a lack of transparency and operational definitions with the investigated performance indicators and issues related to investigating performance indicators over entire competitions. The problems associated with investigating performance indicators without the consideration of contextual and situational factors limit the application of research outcomes into the rugby community.

### Advancing Rugby Performance Analysis

There are some notable studies that have explored the performance processes in rugby union. Recently, researchers have used clustering approaches to identify important patterns in match data associated with certain game outcomes [35, 42]. These methods are useful for reducing large volumes of high-dimensional data to visualisable, low-dimensional output maps or identifying key playing patterns. One method identified that multiple game styles tended to result in success, such as a ball carrying, high-contact style of play. A low possession and strategic kicking style of play was observed to be just as effective. However, it is important to consider that data were not explored in relation to opposition game style for each specific match. This means that support for an ideal game style could not be established. Moreover, the level of competition analysed was low and restricted to a single nation. A *K*-modes cluster analysis was used to identify common playing patterns that preceded a try [42], suggesting plays following lineouts, scrums and kick receipts were common approaches to scoring tries in Super Rugby. A limitation to these approaches is the data related to collective team behaviour, such as player positioning and movements, were not collected in either of these studies.

Multiple studies have considered rugby union performance using a dynamical systems approach to analyse game characteristics [27, 32, 49–55]; however, to the authors’ knowledge, only three studies have used this approach in professional, male adult rugby union contexts [26, 27, 32]. In this approach, important characteristics of complexity are assessed by emergent patterns, due to the interactions between components in the system (i.e. players) over time [51]. This method has been found to successfully identify self-organising, emergent patterns from slight changes in interactions between players [56]. This suggests that players’ decisions and actions are governed not only by prior instruction provided by coaches, but by constraints in the player-environment interaction. In team sports, these behaviours emerge in space and continuously change over time, under the influence of constraints such as task (rules governing the game), environmental (weather) and individual constraints (physical capacity of the athlete) [57], resulting in the spontaneous reorganisations of intrapersonal and interpersonal coordination [58]. Some research has measured the constraining influences of one team on the opposing team’s playing system formation [32]. Attackers were observed to act as a coordinated sub-unit, measured through correlation values, accounting for distance and relative velocity values between each player within the sub-unit (two players from one team) [58]. When the sub-unit of the attacking team was able to disturb the coordination tendencies of the defending team’s sub-unit, this resulted in opportunities for the attacking team to cross the gain line (an imaginary line parallel to the score line, set between the attackers and defenders every time that attackers and defenders perform a ruck, maul, scrum or lineout [32]). However, when both sub-units remained equally coordinated, neither the attacking nor the defending team was successful in crossing the gain line or regaining possession of the ball, respectively. Small adjustments in players’ interpersonal distances and running line speed were considered useful tools to disturb the opponent’s coordination patterns. Using a similar approach, pass decisional behaviour was found to be predicted by the time-to-contact between the attacker and the defender [27]. The type of pass that emerged was significantly correlated (*p* < 0.001) with the variables available in the interaction between players and the environment, suggesting that intrateam coordination is necessary for crossing the gain line as well as effective passing in rugby union.

Capturing movements at the team level associated with successful attacking phases of play, such as advances in territory (achieving a more advanced position in the field of play), have additionally been explored in rugby union [26]. Investigating the multi-player sub-phases, ball displacement trajectory patterns were analysed, revealing the maximum distance the ball travelled backwards from a pass was lower in successful phases of attack. Greater advances in territory were additionally observed when lower backward movements of the ball were coupled with rapid ball delivery. Assessing the macroscopic order therefore suggests successful characteristics in collective behaviour patterns in attacking phases involve a fast ball delivery to a receiver within a close distance [26].

This constraint-led approach is commonly used in the field of skill acquisition and motor learning and proposes novel actions might emerge by manipulating key practice task constraints [51]. This approach has additionally been used to identify the interaction between the intrinsic dynamics and the external constraints within critical match events [27]. Examining the inter- and intrateam coordination patterns that influence successful performance may, therefore, yield critical insights into behaviours associated with successful match events, such as line breaks [22] and try scoring [42]. These methods have yet to be explored in international rugby union and should be addressed in future research.

## Future Direction

A small number of studies have started to progress the field of performance analysis in rugby union [26, 27, 32, 35, 42]. However, compared to various other team sports, the field of dynamical systems analysis in rugby remains largely unexplored. Sports such as football, basketball and AFL have adopted dynamical system approaches in their analysis of tactical performance; however, there is limited understanding of the value of such approaches in a ‘gain line’ team sport, such as rugby union, where teams in possession of the ball aim to gain ground relative to the initial starting position, referenced by a projected line that runs parallel to the try line known as the gain line.

Recognising the need for a multi-dimensional approach to analysing performance, many football researchers have explored the use of novel indicators to assess the tactical behaviour of players [59, 60]. Using positional-derived metrics (such as *x*- and *y*-coordinates), the synchronisation of players’ movements were analysed, revealing positive outcomes associated with time spent synchronised with players from the same team [61]. Variables such as team centre, team dispersion, team interaction and coordination networks and sequential patterns have been explored to generate knowledge about team properties and the patterns that characterise their organisations [62]. These metrics capture intrateam coordination tendencies by measuring the synchronisation of a pair of teammates, known as a dyad, defined as a pair of two players who share the same environment and intentionality, and pursuing common goal-directed behaviours [63]. These dyads form the basis of local social interactions inherent to complex systems, in which individual agents (players) modify their behaviours on the basis of these local interactions and spontaneously organise themselves into coordinated patterns [64]. The local interaction rules are in fact context-dependent, given the presence of other teammates and opponents, demanding the continuous adaptive behaviour of players. Investigators have captured this context dependency through analysing the interpersonal distances between attacker-defender dyads and identifying periods of equilibrium when distances remain a specific distance apart [50]. When interpersonal distance decreases, these systems evolve from a state of balance to critical performance moments, as the contextual dependency rules governing performance require constant co-adaptations of each player to their opponent [50, 51]. It is these local interactions, or system components, governed by their simple local rules, that cause the system to evolve, forming new patterns of dynamics to emerge [51]. By understanding group behaviours and team dynamics during critical performance moments (goal scoring), football analysts are describing the phasic shifts in team dynamics, using team centroids, that can lead to scoring opportunities [65]. Social network theories have also been used to develop a deeper understanding of the passing interactions between team members that demonstrate the local interactions within the wider system [66, 67]. As many of these methods have only been explored in football and basketball, investigating the coordinated patterns of players and continuous interactions as the rugby game evolves is needed to provide a deeper understanding about why certain patterns emerge in critical regions and/or periods in elite-level competition.

Exploring collective system measures and assessing the coordination dynamics between players and teams in elite international level competition may provide valuable insights into team behaviours [68]. This information can then be used to identify patterns of interactions between teammates [62] which coaches can harness to enhance task representation design in training [69].

## Conclusions

The aim of this paper was to critically review the performance analysis research in professional male, 15-a-side rugby union. Studies were assessed based on a number of elements such as context, opposition analysis, competition and number of events analysed.

Studies utilising performance indicators were additionally assessed to establish the genesis of performance indicators and inclusion of operational definitions. Twenty-nine variables were related to successful match outcomes. Possession kicked, lineout success on opposition ball, tries scored, points scored from conversions; tackles completed; turnovers won; and kicks out of hand were the most frequently observed variables. Despite the majority of these articles including context in their analyses, very few accounted for multiple contextual variables, limiting insights into the process of game behaviours due to the player-opponent interaction and the effect of multiple confounding factors, such as field location, number of players involved and period within a match.

Only a third of the studies investigating performance indicators defined the variables used in their analyses. These findings highlight the need for clarity when measuring performance-related variables by providing full operational definitions, to continue to advance the field of performance analysis.

Despite the number of studies published in the last two decades, only a few studies have begun to advance the field, while the majority of the studies reviewed involved a reductionist view of performance. The limited number of studies adopting an alternate view of performance has assessed rugby union performance through a dynamical systems approach by observing emergent patterns. The examination of inter- and intrateam coordination patterns that influence successful performance has the potential to yield critical insights into behaviours associated with successful match events; however, these methods have yet to be explored in international rugby union.

Finally, the advancements in other team sports are discussed to illustrate the potential of a range of performance analysis methods that assess team properties and patterns that characterise their organisation. These methods have been applied to develop a deeper understanding into collective system measures providing valuable insights into sports such as football and basketball.

## Data Availability

Not applicable
